# SIRT7 suppresses the epithelial-to-mesenchymal transition in oral squamous cell carcinoma metastasis by promoting SMAD4 deacetylation

**DOI:** 10.1186/s13046-018-0819-y

**Published:** 2018-07-13

**Authors:** Wenlu Li, Dandan Zhu, Shuaihua Qin

**Affiliations:** Department of Stomatology, The First affiliated hospital of Zhengzhou University, 1# East Jianshe Road 1, Zhengzhou, 450000 Henan China

**Keywords:** sirtuin7, Oral squamous cell carcinoma, Epithelial-to-mesenchymal transition

## Abstract

**Background:**

Oral squamous cell carcinoma (OSCC) is one of the most common malignancies and has a poor prognosis. The epithelial-to-mesenchymal transition (EMT) is crucial for increasing the metastasis of OSCC. Recently, studies have indicated that sirtuin7 (SIRT7) is implicated in tumor genesis; however, the potential role of SIRT7 in the EMT and metastasis of OSCC has not been reported.

**Methods:**

We investigated the cellular responses to SIRT7 silencing or overexpression in OSCC cell lines by wound healing assay, migration and invasion assay, western blotting, immunofluorescence and immunohistochemistry.

**Results:**

In the present study, we found that SIRT7 was significantly downregulated in OSCC cell lines and human OSCC/OSCC tissues with lymph node metastasis. Overexpression of SIRT7 decreased the proliferation and invasion of OSCC cells in vitro, whereas SIRT7 knockdown significantly increased OSCC cell growth and invasion. Upregulation of SIRT7 concomitantly increased the expression of E-cadherin, and decreased the expression of mesenchymal markers. SIRT7 overexpression also reduced the level of acetylated SMAD4 in OSCC cells. Moreover, SIRT7 overexpression significantly inhibited OSCC lung metastasis in vivo.

**Conclusion:**

Together, these findings suggested that SIRT7 suppressed EMT in OSCC metastasis by promoting SMAD4 deacetylation.

## Background

Oral cancer is one of the most common life-threatening types of cancer worldwide, with oral squamous cell carcinoma (OSCC) being the predominant form, accounting for over 90% of all oral malignancies [1–4]. OSCC is associated with high morbidity and mortality [5, 6]. It has been reported that there are over 300,000 new cases of OSCC and 124,000 deaths per year globally [7]. However, despite treatment advances including multi-agent chemotherapy, radiotherapy, and targeted therapy, the overall 5-year survival rate for OSCC remains disappointingly low (< 50%), because of its aggressively invasive and metastatic nature, such that most patients with metastatic disease die within 1 year [8–10]. A better understanding of OSCC pathogenesis and the molecular mechanism underlying its metastasis is therefore needed to inform effective therapies to improve the survival of OSCC patients.

The epithelial-to-mesenchymal transition (EMT) is a vital and fundamental process in the progression and metastasis of OSCC [3, 11–14]. During EMT, epithelial cells gradually lose epithelial structural molecules, polarity, and adhesion capacity, and acquire mesenchymal traits, motility, and invasiveness [1, 15]. Activation of the EMT results in decreased expression of epithelial markers (E-cadherin and β-catenin) and increased expression of adhesion and mesenchymal proteins (including vimentin, N-cadherin and fibronectin) [16, 17]. Transforming growth factor-β (TGF-β) signaling is a key regulator of EMT, which is mediated by SMAD2, SMAD3, and SMAD4. Among several critical factors involved in the complex progression of the EMT, SMAD4, a central transducer of TGF-β signaling, is the most important factor in the clonal evolution and metastases of numerous cancers. Importantly, possible regulators of the SMAD4 pathway in OSCC have rarely been reported, such that further in-depth investigations are merited [18].

Recently, an increasing number of studies have focused on the causal links between aging and cancer metastasis [19, 20]. Sirtuin7 (SIRT7) is the only intranuclear member of the sirtuin family, and is among the most important regulators of aging and lifespan. As a NAD (+)-dependent deacetylase that participates in ribosome biogenesis and rRNA transcription, SIRT7 has been shown to play a prominent role in metabolism, cell cycle control, and proliferation [21–23]. However, at present there is conflicting evidence regarding the role of SIRT7 in tumor genesis and metastasis [19, 23–27]. Some previous studies have reported high expression of SIRT7 in tumors, and especially in high grade tumors, when compared with normal adjacent control tissue [19, 23, 27]. SIRT7 expression has been shown to be negatively correlated with overall survival in patients with colon carcinoma [28]. In contrast to these results, there is controversy regarding whether SIRT7 is truly a cancer-promoting protein [24–26]. McGlynn et al. reported that SIRT7 possesses tumor-suppressing properties in pancreatic cancer, and low levels of SIRT7 expression were associated with an aggressive tumor phenotype and poorer outcomes [29]. Tang et al. reported that SIRT7 was significantly downregulated in breast cancer lung metastases in humans and mice, which highlighted SIRT7 as a suppressor of breast cancer metastasis [24]. In addition, Lai et al. reported that SIRT7 was significantly downregulated in head and neck squamous cell carcinoma (HNSCC) tissues compared with the adjacent noncancerous tissues [25]. However, no study has reported the effect of SIRT7 on OSCC, so the underlying mechanism of this cancer remains elusive.

The present study investigated the role of SIRT7 in OSCC. We evaluated the expression of SIRT7 in human OSCC tissues. Furthermore, the specific effects of SIRT7 on OSCC cell proliferation, migration, EMT, and metastasis, together with the possible underlying mechanisms, were also characterized for the first time.

## Results

### SIRT7 expression is correlated with OSCC progression

**Fig. 1:**
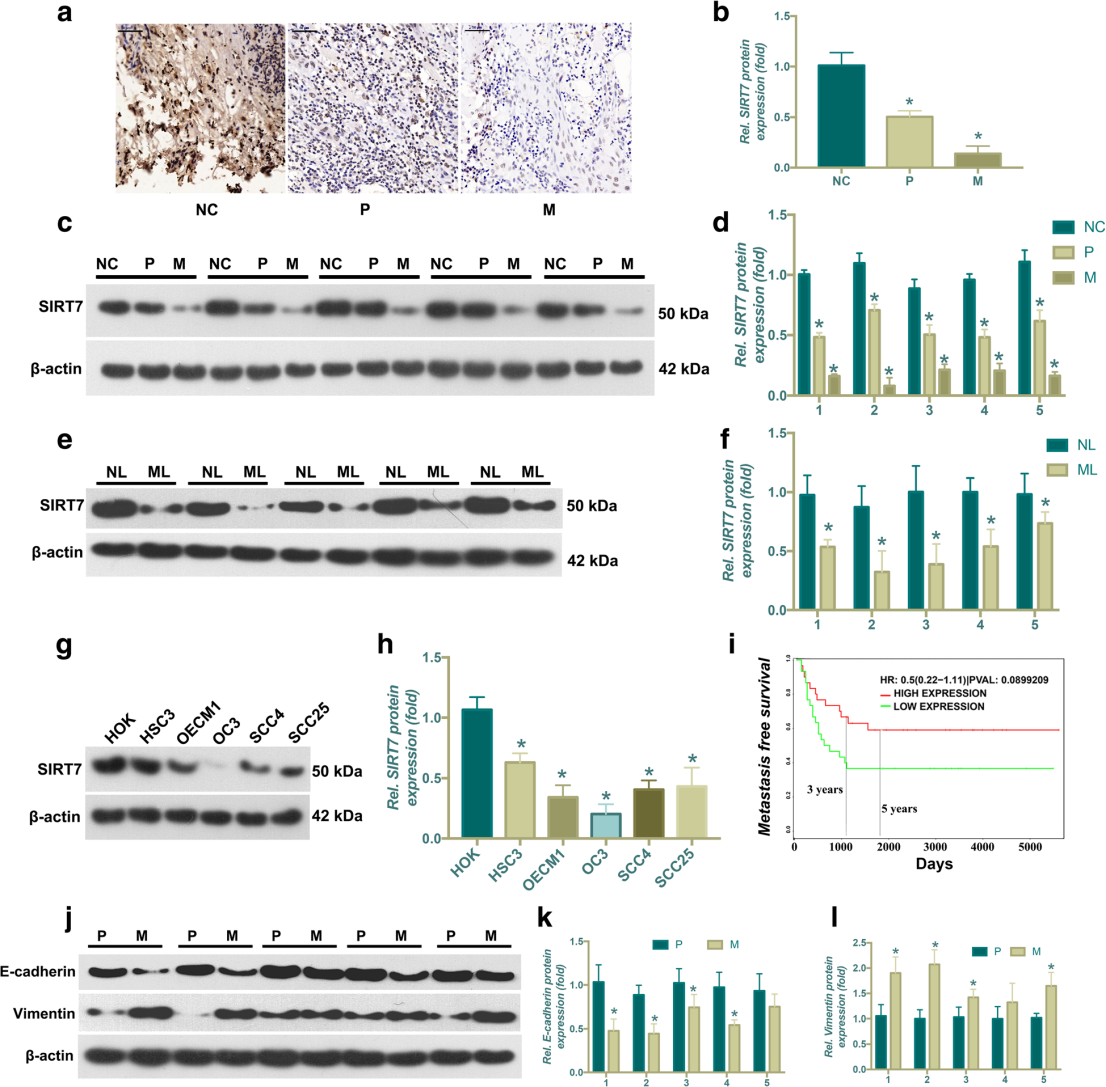
SIRT7 expression is related to OSCC progression*.* (**a**) Representative IHC staining image of OSCC without lymph node metastasis (P, *n* = 20) and OSCC with lymph node metastasis tissues (M, n = 20), and adjacent normal oral tissues (NC, n = 20). (**b**) Relative quantitative comparison of the IHC results of SIRT7 expression in tissue samples between Group P, M, NC; n = 20 each group. * *P* < 0.05 when compared with group NC (**c**) Representative results of WB analysis for SIRT7 protein expression between between Group P, M, NC, *n* = 5 each group. (**d**) Relative quantitative comparison of WB analysis mentioned above. * *P* < 0.05 when compared with group NC (**e**) The results of western blot analysis for SIRT7 protein expression between metastatic lymph nodes tissuess (ML) than the matched primary lymph node (NL). (**f**) Relative quantitative comparison of WB analysis mentioned above. * *P* < 0.05 when compared with group NL; (**g**) The results of western blot analysis for SIRT7 protein expression between HOK and OSCC cell lines. (**h**) Relative quantitative comparison of WB analysis of cell lines. * *P* < 0.05 when compared with the results of HOK cell line.(**i**) The results of bioinformatics analysis based on the ProgeneV2 prognostic database (http://www.abren.net/PrognoScan/). (**j**) Representative results of WB analysis for SIRT7 protein expression between between Group P, M, n = 5 each group. (**k**) Relative quantitative of E-Cadherin levels comparison of WB analysis of the results of P group. * *P* < 0.05 when compared with P group. (**l**) Relative quantitative of vimentin levels comparison of WB analysis of the results of P group. * *P* < 0.05 when compared with P group

We first determined whether SIRT7 expression was associated with human OSCC progression. Immunohistochemical staining was performed on 20 primary OSCC without lymph node metastasis (P) and 20 OSCC with lymph node metastasis (M), and tumor-adjacent normal oral tissues (NC). Representative photographs are shown in Fig. 1a–b. The results showed that there was a significantly lower level of SIRT7 expression in P and M tissues than in NC tissues, which was also confirmed by Western blot (WB) analysis (Fig. 1c–d). The level of SIRT7 were examined by WB analysis between metastatic lymph nodes tissuess (ML) than the matched primary lymph node (NL). The results revealed that a markly higher in matched primary lymph nodes than in metastatic lymph nodes tissues (Fig. 1e–f). Moreover, WB analysis also showed that SIRT7 protein expression was significantly increased in human OSCC cell lines when compared with HOK cells (Fig. 1g–h). To further determine the relationship between SIRT7 expression and human OSCC progression, a bioinformatics analysis based on the ProgeneV2 prognostic database (http://www.abren.net/PrognoScan/) was conducted, which showed that SIRT7 expression correlated inversely with the survival of patients with OSCC, although this correlation did not reach statistical significance (Fig. 1g). In addition, to confirm the status of EMT in metastasis and non-metastasis tumors, E-Cadherin and vimentin were evaluated in primary and metastatic mucosa tissues. The WB analysis shown that higher E-Cadherin levels and lower vimentin levels were in P group compared with those in M group (Fig. 1j–l).

### SIRT7 impairs OSCC cell migration and invasion in vitro

**Fig. 2:**
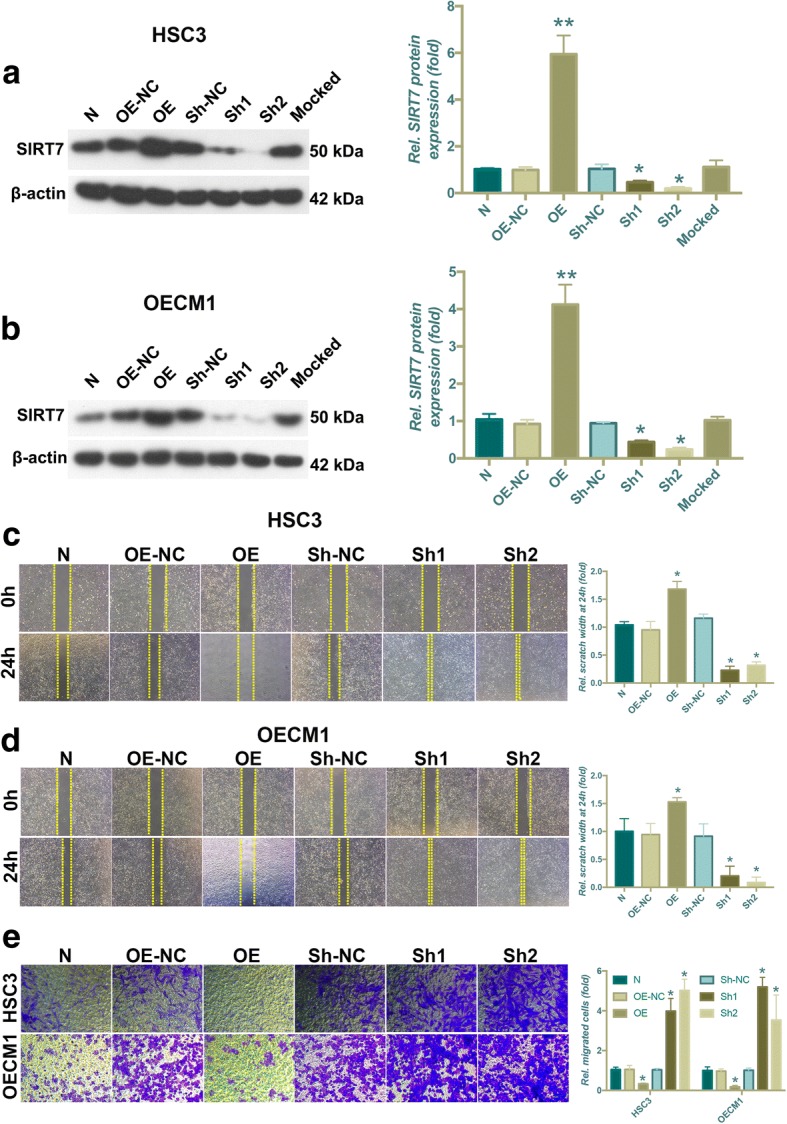
SIRT7 impairs OSCC cells migration and invasion in vitro*.* (**a**) The results of western blot analysis and its quantitative analysis showed successful upregulation and downregulation of SIRT7 levels in HSC3 cells using lentiviral particles. (**b**) The results of western blot analysis and its quantitative analysis showed successful upregulation and downregulation of SIRT7 levels in OECM1 cells using lentiviral particles. (**c**) The results of wound migration assay and quantitative analysis of the effect SIRT7 on HSC3 cells migration. (**d**) The results of wound migration assay and quantitative analysis of the effect SIRT7 on OECM1 cells migration. (**e**) The results of transwell invasion assay and quantitative analysis of the effect SIRT7 on HSC3 and OECM1 cells invasion. N: normal control; OE: overexpression; Sh: shRNA that used for SIRT7 knockdown; NC: negative control. * *P* < 0.05 when compared with group N

To investigate the effects of SIRT7 in OSCC cell lines, lentiviral vectors were used to overexpress and knockdown SIRT7 expression in HSC3 and OECM1 cell lines. As shown in Fig. 2a–b, WB analysis showed that successful SIRT7 overexpression and knockdown were achieved using lentiviral particles. Because SIRT7 expression is related to OSCC progression, SIRT7 may play important roles in one or more steps of OSCC metastasis. We first examined the effects of SIRT7 on OSCC cell migration and invasion. The results of the wound migration assay showed that the wound-healing abilities of SIRT7-overexpressing OSCC cells were significantly lower than those of the control group, while SIRT7 knockdown markedly increased migration (Fig. 2c–d). We further confirmed these findings using a Transwell® invasion assay. Consistent with results obtained from the wound-healing assay, lentiviral shRNA-mediated SIRT7 silencing significantly increased the invasion of OSCC cells. In contrast, SIRT7 overexpression decreased the invasion of OSCC cells (Fig. 2e).

### SIRT7 suppresses the EMT in OSCC cells

**Fig. 3:**
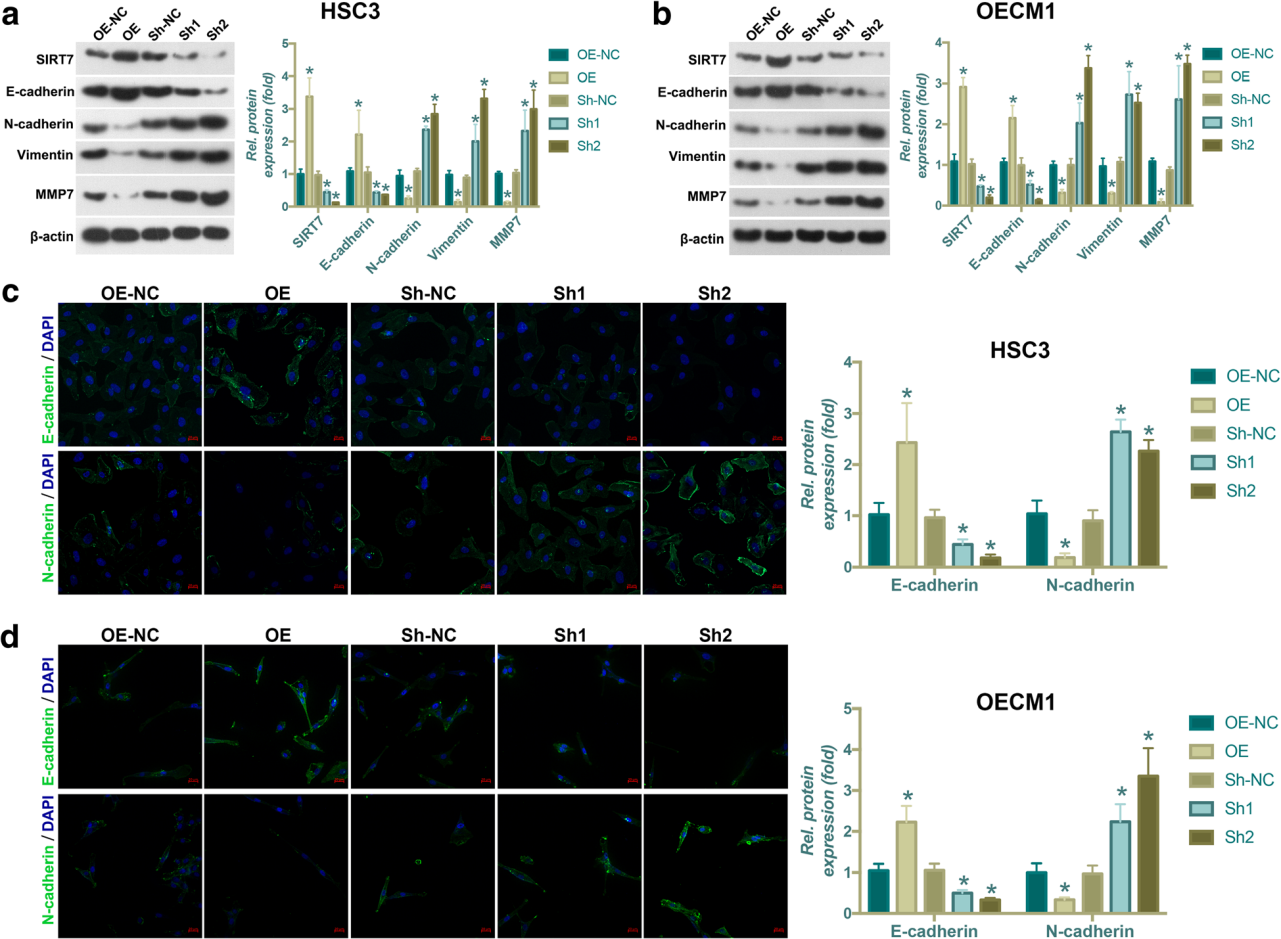
SIRT7 suppresses EMT in OSCC cells. (**a**) The results of western blot analysis and its quantitative analysis of the effects of SIRT7 on EMT makers in HSC3 cells. (**b**) The results of western blot analysis and its quantitative analysis of the effects of SIRT7 on EMT makers in OECM1 cells. (**c**) The results of immunofluorescence staining and its quantitative analysis of the effects of SIRT7 on E-cadherin and N-cadherin protein expression in HSC3 cells. (**d**) The results of immunofluorescence staining and its quantitative analysis of the effects of SIRT7 on E-cadherin and N-cadherin protein expression in OECM1 cells. OE: overexpression; Sh: shRNA that used for SIRT7 knockdown; NC: negative control. * *P* < 0.05 when compared with group Sh-NC

To identify the molecular mechanisms involved in SIRT7-mediated regulation of cell migration and invasion in OSCC cell lines, an analysis of the main EMT markers was performed. As shown in Fig. 3a–b, the results of WB analysis showed that SIRT7 overexpression dramatically increased E-cadherin expression and downregulated the protein levels of N-cadherin, vimentin, and MMP7. In contrast, lentiviral shRNA-mediated SIRT7 silencing significantly decreased E-cadherin expression and upregulated the protein levels of N-cadherin, vimentin, and MMP7. The effects of SIRT7 on E-cadherin and N-cadherin were further confirmed by immunofluorescence staining (Fig. 3c–d). Taken together, these results showed that SIRT7 overexpression suppressed the EMT in OSCC cells.

### SIRT7 suppresses the EMT in OSCC cells by deacetylating SMAD4

**Fig. 4:**
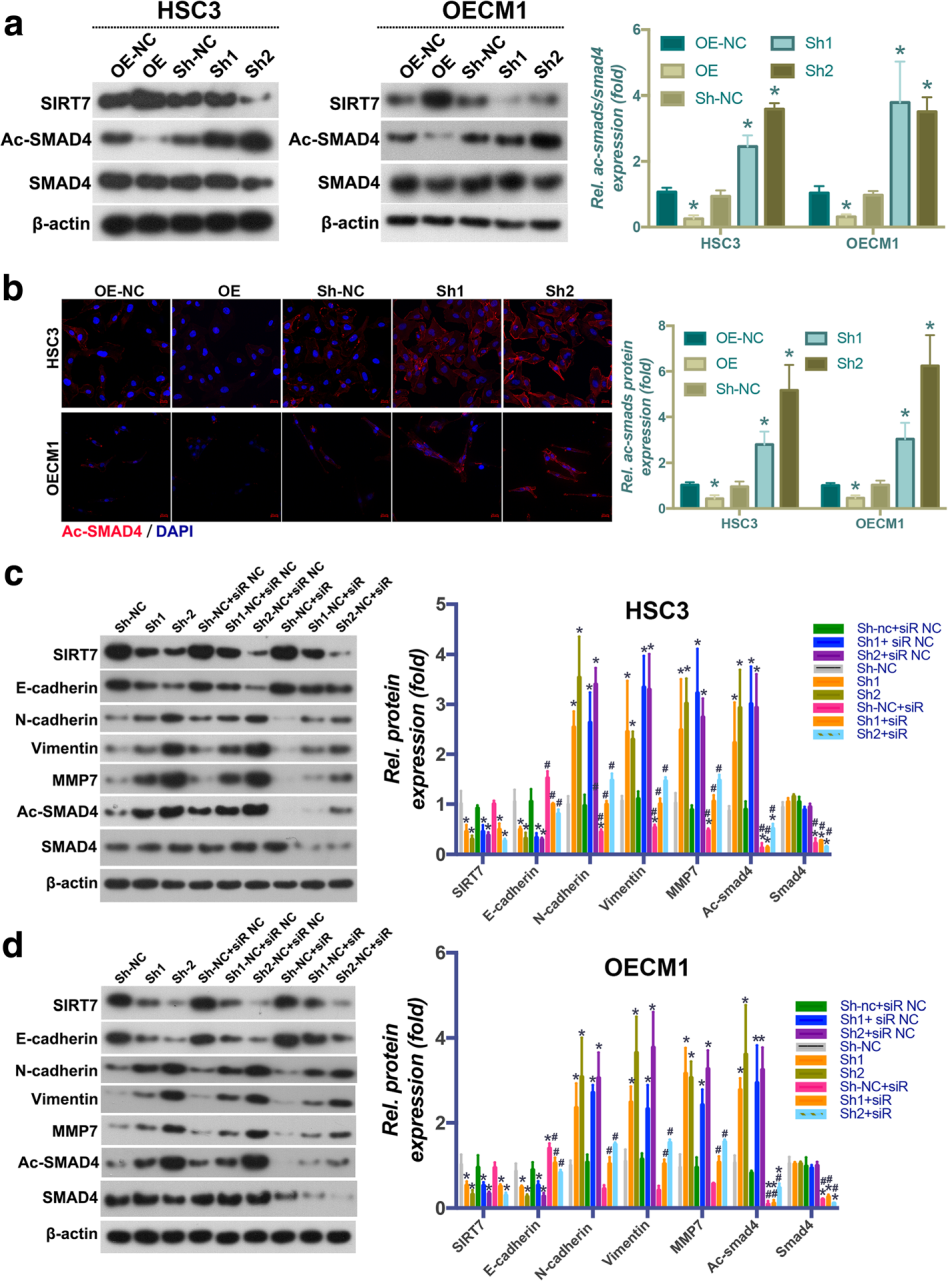
SIRT7 suppresses EMT in OSCC cells through deacetylating SMAD4. (**a**) The results of western blot analysis and its quantitative analysis of the effects of SIRT7 on SMAD4 and Ac-SMAD4 protein expression in HSC3 and OECM1 cells. (**b**) The results of immunofluorescence staining and its quantitative analysis of the effects of SIRT7 on Ac-SMAD4 protein expression in HSC3 and OECM1 cells. (**c**) The results of western blot analysis and its quantitative analysis of the effects of SIRT7 on SMAD4, Ac-SMAD4 and EMT markers protein expression in HSC3 cells. (**d**) The results of western blot analysis and its quantitative analysis of the effects of SIRT7 on SMAD4, Ac-SMAD4 and EMT markers protein expression in OECM1 cells. OE: overexpression; Sh: shRNA that used for SIRT7 knockdown; NC: negative control. * *P* < 0.05 when compared with group OE-NC; # *P* < 0.05 when compared with group siR NC groups

To identify the molecular mechanism underlying the effects of SIRT7 on the EMT in OSCC cells, components of the SMAD4 signaling pathway, a key regulator of the EMT, were evaluated. As shown in Fig. 4a, WB analysis showed that SIRT7 overexpression significantly promoted SMAD4 deacetylation; the acetylated (Ac)-SMAD4 protein expression level was decreased during SIRT7 overexpression. In contrast, SIRT7 knockdown increased the protein level of Ac-SMAD4, which was further confirmed by immunofluorescence staining (Fig. 4b). After application of a siRNA that targeted SMAD4, the effect of SIRT7 knockdown on increasing Ac-SMAD4 expression was significantly reduced. In addition, the effect of SIRT7 knockdown on increasing the EMT in OSCC cells was also significantly inhibited (Fig. 4c–d). Taken together, these results suggested that the suppressive effect of SIRT7 on the EMT in OSCC cells was at least partially mediated by promotion of SMAD4 deacetylation.

### SIRT7 overexpression inhibits metastasis in vivo

**Fig. 5:**
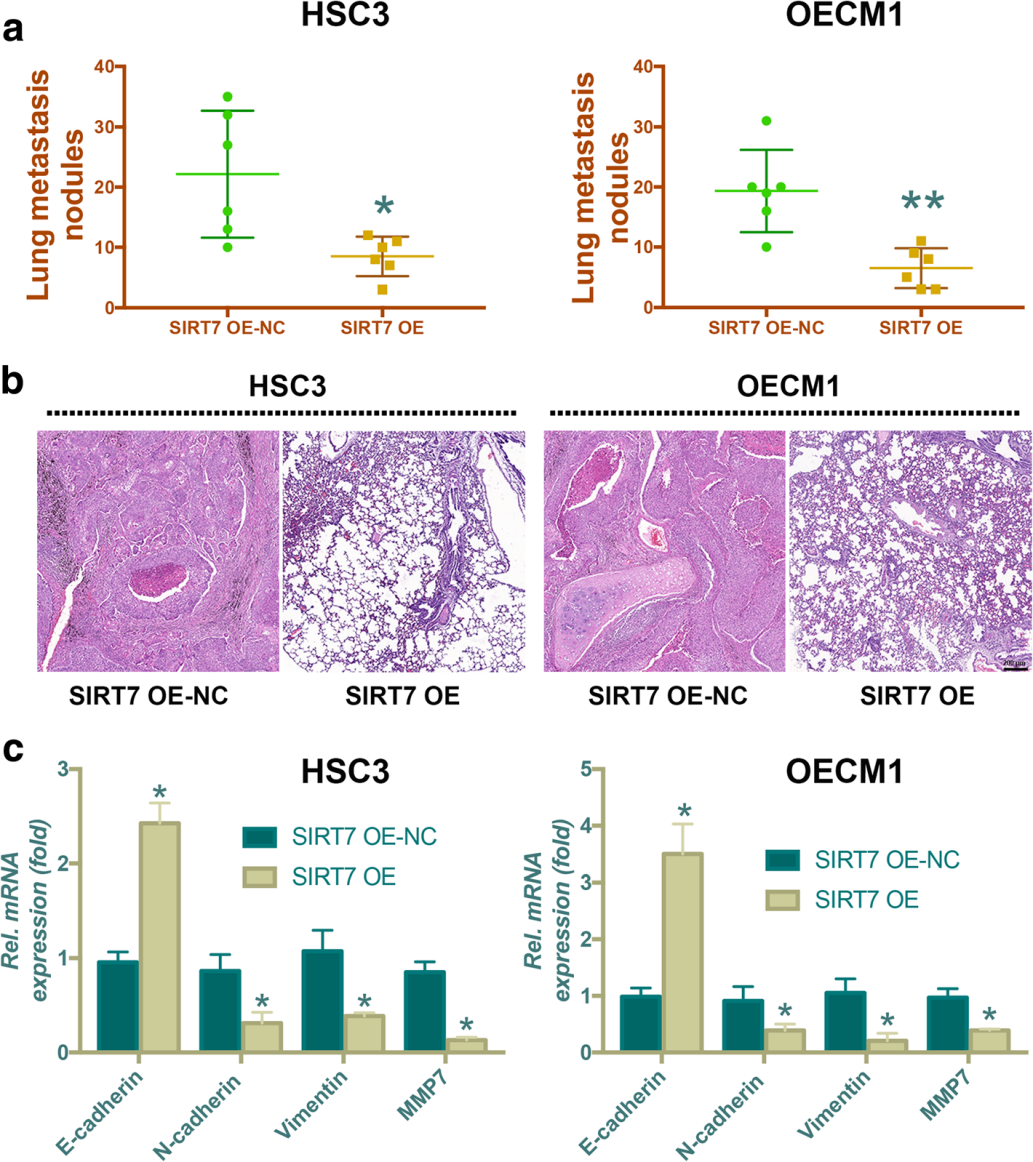
SIRT7 overexpression inhibits metastasis in vivo. (**a**) Scatter plot showing lung metastatic nodules (*n* = 6 for each group). (**b**). Representative hematoxylin and eosin (HE) images for OSCC metastatic lung nodules. (**c**) mRNA levels of E-cadherin, N-cadherin, Vimentin, and MMP7 of each group. OE: overexpression; NC: negative control. * *P* < 0.05 when compared with group OE-NC

The in vivo anti-metastatic effects of SIRT7 on OSCC were determined using a tail vein metastatic assay. Compared with mice injected with SIRT7-overexpressing HSC3 and OECM1 cells, more nodules in the lung were observed in mice that were injected with empty virus vector-infected HSC3 and OECM1 cells (Fig. 5a–b). Hematoxylin and eosin staining showed that tail vein injection with cells stably overexpressing SIRT7 led to significantly fewer and smaller nodules in lungs compared with the control group (Fig. 5c–d). In addition, increased E-cadherin and downregulated N-cadherin, vimentin, and MMP7 mRNA levels were observed in the lung tissue of the SIRT7 overexpression group. Taken together, these results showed the remarkable inhibitory effects of SIRT7 overexpression on pulmonary metastasis of OSCC cells.

## Discussion

We have shown that SIRT7, a newly discovered and pivotal regulator of lifespan and aging, acted as a tumor metastasis-suppressing gene in OSCC, as shown by its downregulation promoting OSCC cell line invasion and migration (whereas its overexpression suppressed metastasis). The decreased expression of SIRT7 in primary OSCC tissues without metastatic lymph node and OSCC tissues with metastatic lymph node indicated that it may be useful for predicting the prognosis of OSCC patients, as well as the likelihood of relapse and metastasis. We further found that SIRT7 overexpression-induced metastasis suppression was dependent on its negative regulation of the EMT, at least partially through promoting SMAD4 deacetylation, because its upregulation induced resistance to the EMT, whereas its deletion attenuated the EMT and metastasis in OSCC.

This study emphasized the novel and necessary role of SIRT7 in tumor metastasis. Although previous reports studied the role and function of SIRT7 in various cancers, they mainly focused on tumor growth (e.g., cell proliferation, apoptosis, and cell cycle arrest). Furthermore, the results of these studies were controversial: some reported that SIRT7 was responsible for the maintenance of oncogenic transformation via H3K18ac deacetylation [30]. Yu et al. reported that overexpression of SIRT7 had oncogenic properties and served as a prognostic factor in colorectal cancer [28]. Similar results were also reported by Malik et al. [31]. However, Vakhrusheva et al. reported that as an anti-aging factor, SIRT7 was consistently downregulated in tumorigenic cells. SIRT7 is thought to play an important role in mediating tissue integrity during aging by inhibiting cell growth and proliferation [32]. Lai et al. analyzed SIRT genes in human HNSCC tissues (including OSCC) from 39 patients and reported that SIRT7 was negatively correlated with the progression of human HNSCC [25]. Because of these controversial findings, Tang et al. [24] conducted an in depth study of breast cancer and reported that SIRT7 may itself be dynamically regulated during tumor development, which means that at an early stage, high SIRT7 could contribute to oncogenic transformation and tumor growth while, later, it inhibits migration and invasion of tumor cells. Although the results of our study were consistent with their findings, future studies focusing on dynamic changes in SIRT7 role during tumorigenesis are still required.

There is presently little known about the potential role of SIRT7 in cancer metastasis. Our results showed that upregulation of SIRT7 significantly impaired the invasion and migration of OSCC, both in vitro and in vivo, but its knockdown dramatically promoted OSCC metastasis. A recent report by Tang et al. [24] also indicated that upregulation of SIRT7 antagonized the EMT of breast cancer and inhibited its metastasis. To some extent, this study further supported our findings that SIRT7 plays an important role in tumor invasion, migration, and metastasis.

The EMT has widely been considered as a vital process in regulating the initial steps in OSCC metastasis. Because of its clinical importance, suppression of the EMT is becoming an attractive therapeutic strategy that can significantly improve the disease outcome of cancer patients. Our findings implicated SIRT7 as a key regulator of the EMT in OSCC, where overexpression of SIRT7 induced a range of biochemical (increased E-cadherin and decreased N-cadherin and vimentin) and functional (decreased MMP9 production, and decreased invasion and migration capacity) changes. TGF-β signaling is a key regulator of the EMT, which is mainly mediated by SMAD4. However, little is known regarding the possible correlation between the regulation of SMAD4 and the EMT of OSCC. Consistent with previous studies, we found that SIRT7 deacetylated and promoted SMAD4 degradation [24]. In another study, Chen et al. reported that by deacetylating SMAD4, the SIRT1 enzyme can also reduce MMP7 expression and consequently inhibit cell migration, invasion, and tumor metastasis in OSCC [18].

Loss of SIRT1 has been reported to cause hyper-acetylation of SMAD4 and promote breast cancer metastasis [33]. Together with recently reported studies about the interaction between SIRT1 and SIRT7 during gene transcription [31], the impact of SIRT7 on the EMT may also be closely regulated by SIRT1, but further studies are needed to confirm this possibility.

Although additional future studies are needed to identify the detailed mechanism, our results indicated that SIRT7 suppressed the EMT in OSCC metastasis by promoting SMAD4 deacetylation, which constitutes an important insight into the role of SIRT7 in tumor metastasis.

## Methods

### Clinical tissues

This study was approved by the Medical Ethics Committee of the First affiliated hospital of Zhengzhou University Hospital. All subjects provided written informed consent, and none of them received chemo radiotherapy prior to surgery. Clinical tissue samples of OSCC and the paired adjacent normal tissue, together with lymph node metastasis samples were obtained during surgery. The relevant clinical information were in Table 1.

**Table 1:** The clinical profiles of the 40 patients with OSCC

Clinical parameters	No. of patients		*P* value
	Metastatic (n = 20)	Nonmetastatic (*n* = 20)	
Gender			
Female	12	10	> 0.05
Male	8	10	
Age			
mean ± SD	51.23 ± 11.63	56.82 ± 15.59	> 0.05
Lymph node status			
N-	0	20	< 0.001
N+	20	0	
Tumor classification			
T1 + T2	2	13	< 0.05
T3 + T4	18	7	

### Bioinformatics analysis

The ProgeneV2 prognostic database (http://www.abren.net/PrognoScan/) was used to collect information for analysis of the effect of SIRT7 on survival in OSCC patients [34, 35]. Kaplan-Meier curve was applied for analyzing survival rate of patients with OC.

### Immunohistochemistry (IHC)

IHC staining was performed in paraffin-embedded specimens a blind manner as follows: the slides were incubated overnight with rabbit anti-SIRT7 monoclonal antibody (1:100; Abcam, Cambridge, UK) employing an avidin-biotin complex method. A secondary antibody was then applied for 30 min at room temperature.

### Cell culture

The HOK cells and five human OSCC cell lines (HSC3, OECM1, OC3, SCC4, and SCC25 used in this study were purchased commercially from the American Type Culture Collection (ATCC; Manassas, VA, USA). The HOK cells were cultured in oral keratinocyte growth medium (ScienCell, Carlsbad, CA, USA), HSC-3 and OC3 cells were cultured in DMEM) medium, OECM-1 cells were maintained in RPMI 1640 medium, while SCC4 and SCC25 cells were cultured in DMEM/F12 medium. All the medium mentioned above were supplemented with 10% fetal bovine serum (FBS; Hyclone, Israel) and 100 units/mL penicillin and streptomycin (P/S, Invitrogen, Camarillo, CA, USA). Cells were maintained at 37 °C in a atmosphere filled with 5% CO2.

### Immunofluorescence staining

For Immunofluorescence staining, cells were grown onto coverslips and fixed in 4% paraformaldehyde for 10 min, and blocked with in 0.05% Triton X-100 and 3% bovine serum albumin (BSA) for 30 min. The coverslips were incubated primary antibodies (N-cadherin (1:100; Abcam, Cambridge, United Kingdom), E-cadherin (1:100; Abcam, Cambridge, United Kingdom), acetylated smad4 (1:100; Proteintech, Wuhan, China)) overnight at 4 °C, followed by fluorescence-conjugated secondary antibodies (Beyotime) for 2 h at room temperature, and nuclei were stained with DAPI (Beyotime) for 5 min.

### Cell transfection and RNA interference

A lentiviral short hairpin RNA (shRNA) construct targeting SIRT7 was obtained from Sigma-Aldrich (St. Louis, MO, USA). For stable knockdown, the shRNAs were annealed, and cloned into the pLKO.1 vector (Sigma). The SIRT7 overexpression particles were purchased by GenePharma (Shanghai, China). For retroviral overexpression of SIRT7, polymerase chain reaction (PCR) was used to obtain SIRT7 cDNA, which was then subcloned into the BamHI and XhoI sites of the LV3 retroviral vector. Lentiviral transfection was performed strictly according to the manufacturer’s protocols. In brief, OSCC cells (HSC3 and OECM1 cells) were seeded at 2 × 10^5^ cells/well in a 6-well plate before transfection and incubated with 2 ml of complete medium for 24 h. Cells were then transfected with lentiviral particles for 12 h, after that the virus-containing medium was replaced with fresh complete medium. Finally, cells were selected using 4 μg/ml puromycin for 96 h to get OSCC cells with stable SIRT7 overexpression or knock-down. Empty lentiviral vectors were used as a control. siRNA transfection targeting SMAD4 was performed with Lipofectamine 2000 (Thermo) following the manufacturer’s instruction, using siRNAs synthesized in GenePharma (Shanghai, China).

### RNA isolation and quantitative real-time PCR (RT-qPCR)

Total RNA was isolated using TRIzol reagent (Takara, Dalian, China), which was then reversely transcribed into cDNA using PrimeScript RT-PCR kit (TaKaRa Biotechnology) according to the protocol. The RT-qPCR was performed using the Power SYBR Green PCR Master Mix on the ABI 7900 Prism HT (Applied Biosystems). Primers used are followed: E-cadherin, Forward: CGAGAGCTACACGTTCACGG; Reverse: GGGTGTCGAGGGAAAAATAGG; N-cadherin, Forward: TTTGATGGAG.

GTCTCCTAACACC; Reverse: ACGTTTAACACGTTGGAAATGTG; Vimentin, Forward: GCCCTAGACGAACTGGGTC; Reverse: GGCTGCAACTGCCTAATGA.

G; MMP7, Forward: GAGTGAGCTACAGTGGGAACA; Reverse: CTATGACGC.

GGGAGTTTAACAT; GAPDH, Forward: TGTGGGCATCAATGGATTTGG; Reverse: ACACCATGTATTCCGGGTCAAT. The 2^−△△Ct^ method was used to calculate relative gene expression.

### Wound healing assay

The wound healing assay was performed to test the effect of SIRT7 in OSCC cell migration. OSCC cells (HSC3 and OECM1 cells) with SIRT7 overexpression and knock-down together with the control groups were cultured in into 12-well plates and cultured to 90% confluence. The cells were then starved overnight, and the wound was scratched in the center of the cell monolayer using a sterile 1-mL micropipette tip. The wound was photographed under a microscope (DM IL, Leica Microsystems) at indicated time (0 h and 24 h after the wound was created). Image J sofware was used to quantify the relative migration rate.

### Transwell invasion assay

The transwell invasion assay was performed to test the effect of SIRT7 in OSCC cell invasion. After transfection, 5 × 10^4^ OSCC cells (HSC3 and OECM1 cells) suspended in medium without FBS were incubated on the upper chamber membranes using a BioCoat Matrigel Invasion Chamber (Corning) strictly according to the Cell Invasion Assay protocol. The lower chamber was incubated in 500 μl medium with 10% FBS. To evaluate the invasive ability of OSCC cells, non-invasive cells were removed by cotton swabs and invasive cells were stained with crystal violet and counted under a microscope (DM IL, Leica Microsystems).

### Western blot (WB) analysis

Protein extracts from OSCC cells and clinical tissue samples were prepared in radioimmunoprecipitation assay lysis buffer supplemented with a protease inhibitor (Beyotime, Haimen, China). Protein samples were separated by 10% SDS polyacrylamide gels and then transferred to a PVDF membrane (Millipore). After blocking in 5% BSA for 1 h, the membranes were incubated overnight at 4 °C with primary antibodies specific to β-actin (1:2000; CST), N-cadherin (1:1000; CST), E-cadherin (1:1000; CST), SIRT7 (1:1000; CST), MMP7(1:1000; CST), SMAD4 (1:1000; CST), Vimentin (1:1000; CST), and acetylated SMAD4 (1:100; Proteintech, Wuhan, China), which was then probed with a horseradish peroxidase-conjugated goat anti-rabbit IgG (1:1000) as a secondary antibody for 2 h at room temperature. β-actin was served as the internal control for all western blots. Immunoblots were visualized and quantified using the Bio-Rad system (Bio-Rad, Hercules, CA, USA).

### Xenograft model of OSCC lung metastasis

Animal studies were approved and conducted strictly in accordance with institutional ethical guidelines the Committee on the Use of Live Animals in Teaching and Research of Zhengzhou University. Briefly, 4-week-old pathogen-free female BALB/c-nu mice were purchased from the Slaccas (Shanghai), which were housed in SPF barrier facilities under a 12 h light/dark cycle, and supplied with sterilized food and water. For in vivo xenograft studies, 24 mice were randomly selected and divided into four groups. On day 0, 1 × 10^6^ HSC3^OE-NC, HSC3^sirt7 OE, OECM1^OE-NC, OECM1^SIRT7 OE cells suspended in 0.1 ml of PBS were injected via tail vein of each mouse (six mice in each group). Mice were killed at 8 weeks after tail vein injection, lungs were collected and stored in liquid nitrogen or fixed in formalin for further analysis. The number of metastatic nodules on lung surface was counted under dissecting microscope after hematoxylin and eosin (H&E) staining.

### Statistical analysis

All experiments were carried out with at least three replicates. The data were shown as mean ± S.D. One-way ANOVA with Dunnett’s test or Newman Keuls test and Student’s two-tailed t-test were used to calculate the significant differences. Differences were considered as statistically significant when *P* < 0.05.

## Conclusions

In conclusion, the present study showed that SIRT7 overexpression suppressed cell migration and invasion in OSCC. Furthermore, SIRT7 suppressed the EMT of OSCC, at least partially by promoting SMAD4 deacetylation. Our study also showed the functional significance of SIRT7 upregulation in metastatic OSCC.

## Abbreviation

EMT: epithelial-to-mesenchymal transition
OSCC: oral squamous cell carcinoma
SIRT7: sirtuin7
SMAD: drosophila mothers against decapentaplegic protein
TGF-β: transforming growth factor-β
WB: western blot
